# Efficacy and Safety for the Use of Half-Dosed Pegylated Granulocyte Colony-Stimulating Factors in Preventing Febrile Neutropenia During Chemotherapy in Patients With Malignant Tumors: A Multicenter, Open-Labeled, Single-Arm Phase 2 Trial

**DOI:** 10.3389/fonc.2022.820324

**Published:** 2022-04-27

**Authors:** Qi Mei, Xiaoyu Li, Runkun Wang, Kai Qin, Yi Cheng, Weiting Cheng, Youhong Dong, Zhen He, Jun Li, Ming Li, Xi Tang, Xudong Wang, Xuxuan Xiao, Bin Yang, Yajuan Zhou, Rui Wang, Qiao Huang, Guangyuan Hu, Jian Li

**Affiliations:** ^1^Department of Oncology, Tongji Hospital, Tongji Medical College, Huazhong University of Science and Technology, Hubei, China; ^2^Department of Oncology, the First People’s Hospital of Guangshui, Hubei, China; ^3^Department of Oncology, Wuhan No. 1 Hospital, Wuhan, China; ^4^Department of Oncology, Xiangyang No. 1 People’s Hospital, Affiliated Hospital of Hubei University of Medicine, Hubei, China; ^5^Department of Internal Medicine, Henan Cancer Hospital, Affiliated Cancer Hospital of Zhengzhou University, Henan, China; ^6^Department of Oncology, Xiaogan Central Hospital, Xiaogan, China; ^7^Department of Oncology, Wuhan Pulmonary Hospital, Wuhan, China; ^8^Department of Oncology, Jingzhou Central Hospital, Hubei, China; ^9^Department of Oncology, Huangshi Central Hospital, Huangshi, China; ^10^Department of Oncology, Hubei Cancer Hospital, TongJi Medical College, Huazhong University of Science and Technology, Hubei, China; ^11^Department of Radiation Oncology, Hubei Cancer Hospital, TongJi Medical College, Huazhong University of Science and Technology, Hubei, China; ^12^Department of Oncology, The First College of Clinical Medical Science, China Three Gorges University, Yichang, China; ^13^Institute of Molecular Medicine and Experimental Immunology, University Clinic of Rheinische Friedrich-Wilhelms-University, Bonn, Germany

**Keywords:** febrile neutropenia, chemotherapy, phase 2 trial, half dose of PEG-rhG-CSF, adverse effect, multiple cancers, prophylactic use

## Abstract

**Background:**

Prophylactic granulocyte-colony stimulating factor (G-CSF) has been shown to effectively prevent febrile neutropenia (FN) and grade 3/4 neutropenia during myelosuppressive treatment. The present study reports the clinical efficacy and safety of the prophylactic use of G-CSF with a half dose for cancer patients with an intermediate risk of FN combined with ≥1 patient-specific risk during multiple chemotherapy.

**Methods:**

This multicenter, one-arm, and open-label clinical study involved 151 patients [median age, 54 years old (range, 46.0–62.5); 38.4% female] with malignant tumors, including >20 different cancers. These patients underwent a total of 604 cycles of chemotherapy and received a half dose of PEG-rhG-CSF administration prior to each cycle.

**Results:**

The incidence rate of FN was 3.3% for this cohort during chemotherapy. Chemotherapy delay occurred in 6 (4.0%) patients for 12 (2.0%) cycles. Early termination of cancer treatment occurred in 14 (9.3%) patients. In this cohort, 23 (15.2%) patients required antibiotic use during courses of chemotherapy. A total of 28 (18.5%) patients experienced clear adverse effects during cancer treatment.

**Conclusion:**

The prophylactic PEG-rhG-CSF with a half dose can both efficaciously and safely prevent neutropenia for patients of diverse cancers with an intermediate risk of FN combined with ≥1 patient-specific risk during chemotherapy.

## Introduction

The incidence of febrile neutropenia (FN) is the major adverse effect for cancer patients during myelosuppressive chemotherapy (1). Development of FN can lead to increase in treatment cost, delay, or prolongation of treatment, and eventually dramatic reduction in treatment efficacy (2). According to current National Comprehensive Cancer Network (NCCN) guidelines, patients during a course of chemotherapy at a risk of developing FN ≥ 20%, 10% ≤ FN < 20%, and FN <10% are considered high, intermediate, and low risk, respectively. In the past, the prophylactic use of granulocyte-colony stimulating factor (G-CSF) drugs such as filgrastim (3) and pegfilgrastim (4) has been frequently applied for patients at a high or intermediate risk during chemotherapy of various cancers and shown to greatly reduce the incidence of FN (5, 6). However, the adverse effects of G-CSF drugs consist of bone pain, bleomycin-induced pulmonary toxicity, and/or other toxicities, which could eventually lead to lethal case, increasing the mortality rate of G-CSF drugs (7, 8). Recent studies have demonstrated with patient cohorts of multiple cancer types that prophylactic use of pegylated recombinant human G-CSF (PEG-rhG-CSF) could reduce the incidence rate of FN to <8% during intense dose chemotherapy, compared to the incidence rate of FN >20% without the use of a G-CSF drug (9, 10). Current NCCN guidelines recommend that patients with a high risk and intermediate risk combined with ≥1 patient-specific risk factors for FN should prophylactically use a G-CSF drug with the same full dose during the course of chemotherapy. This may not fully reflect the purpose of precision medicine, since patients with an intermediate risk might already benefit from the reduced dose of G-CSF. Additionally, several clinical trials provided indications for the efficacy of G-CSF drugs with half dose on cancer patients (11–13). Thus, it is necessary to investigate this issue given the high demand and frequent use of G-CSF drugs. The present study intended to investigate whether the prophylactic use of a G-CSF drug with a half dose in cancer patients with an intermediate risk combined with ≥1 patient-specific risk factors for FN would achieve satisfactory efficacy and safety effect during multiple cycles of chemotherapy. The results of the present study might help stakeholders and health systems effectively allocate their resources.

## Patients and Methods

**Figure 1:**
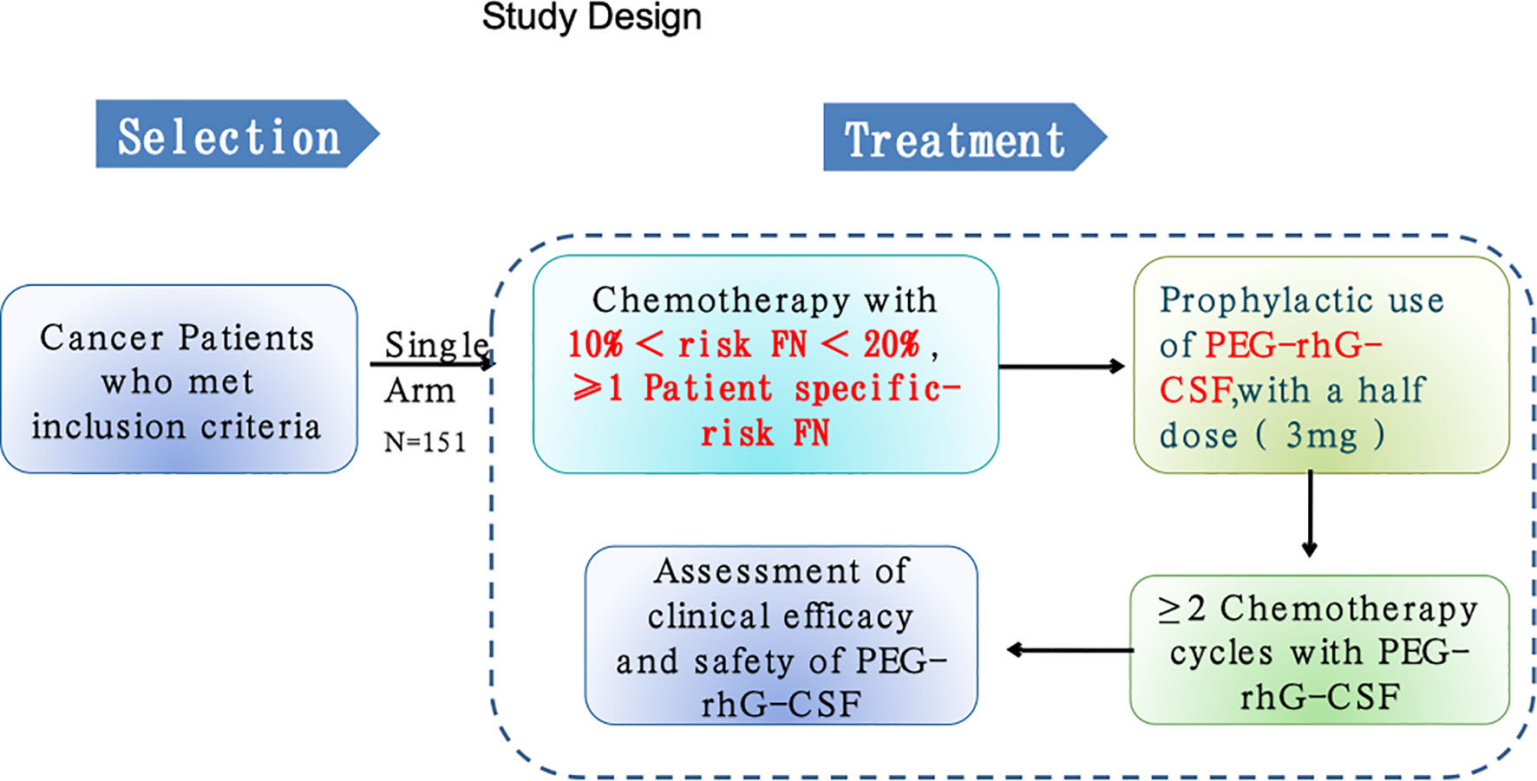
The chart of study design.

This was a phase 2, multicenter, open-labeled, single-arm trial. Adult patients aged at least 18 years with confirmed diagnosis of malignant tumors and required multiple-cycle chemotherapy from one of 11 hospitals in China from April 2018 to November 2019 were recruited for this study (**Table 1**; **Figure 1**). All participants provided written informed consent for protocol-based treatment. The present study was conducted in accordance with the Declaration of Helsinki. The protocol has been approved by the China Ethics Committee of Registering Clinical Trials (No. ChiECRCT-20180146) and registered in the Chinese Clinical Trial Registry (No. ChiCTR1800019675).

**Table 1:** Patient and tumor characteristics and involved hospitals (N = 151).

Characteristics	No. (%)/median (IQR)
Age (year)	54 (46.0–62.5)
Gender (female)	58 (38.4)
Hight (mm)	165.0 (159.0–171.0)
Weight (kg)	60.0 (53.0–68.5)
Body surface area (m^2^)	1.69 (1.56–1.81)
Surgery	
Yes	86 (57.0)
No	65 (43.0)
Comorbidity status	
Yes	110 (72.8)
No	41 (27.2)
Cancer type	
Lung	38 (25.2)
Head and neck	26 (17.2)
Breast	22 (14.6)
Intestinal track	18 (11.9)
Lymphoma	11 (7.3)
Others	36 (23.8)
T stage	
I	0
II	0
III	70 (46.4)
IV	81 (53.4)
Involved Hospitals	Recruited patients
Tongji Hospital	90 (59.6)
Yichang Hospital	9 (6.0)
Xiaogang Hospital	6 (4.0)
Wuhan Pulmonary Hospital	4 (2.6)
Huangshi Hospital	4 (2.6)
Guangshui Hospital	24 (15.9)
Xiangyang Hospital	1 (0.7)
Wuhan Cancer Hospital	9 (6.0)
Wuhan No. 1 Hospital	1 (0.7)
Jingzhou Hospital	2 (1.3)
Henan Cancer Hospital	1 (0.7)

### Patient Eligibility

Inclusion criteria were (1) age of at least 18 years old; (2) confirmed diagnosis of malignant tumor by histopathological or cellular analysis; (3) requirement for multiple-cycle chemotherapy; (4) estimated FN risk between 10% and 20% (intermediate risk group), combined with at least one of the following patient-specific risk factors including previously experienced chemo- or radiotherapy, durable FN, tumor affecting bone marrow, recent operation or open wound, liver dysfunction with bilirubin >2.0, kidney dysfunction with creatinine clearance <50, and received dose-intensive chemotherapy with an age older than 65 (14–16); (5) Karnofsky performance score (KPS) ≥70; (6) estimated survival >3 months; (7) normal function of bone marrow (ANC ≥ 1.5×10^9^/L, PLT ≥ 80×10^9^/L, Hb ≥ 75g/L, WBC ≥ 3.0×10^9^/L); (8) agreement to effective contraception during the study period and 6 months after discontinuation of treatment; and (9) willingness to participate.

Exclusion criteria were (1) received stem-cell and/or organ transplantation, (2) inadequately controlled local or systemic infections, (3) severe internal organ dysfunction and myocardial infarction in the past 6 months, (4) liver functional-related laboratory measurements such as TBIL, ALT, and AST all twofold above the threshold of normal range, in case of liver metastasis, fivefold above the threshold; or creatinine clearance is twofold above the threshold of normal range, (5) spleen hyperfunctionality, (6) interval from last participation in other clinical trials to the present study is <4 weeks; (7) allergic to the PEG-rhG-CSF and rhG-CSF, (8) severe mental illness that can affect the decision-making for participate in this study, (9) pregnant or lactating female patients or those who refuse to accept contraception, and (10) patients who received concomitant radiation therapy; (11) patients who were determined as not quantified or suitable to participate in this study. Criteria for exit from the study were (1) occurrence of adverse events that patients cannot tolerate, (2) tumor progression, and (3) withdrawal of participation consent. FN was defined using NCCN criteria as ANC <1.0 × 10^9^/L, and the duration of body temperature >38°C lasts longer than 1 h. In the present study based on our empirical experience, an FN that lasted to a next cycle of chemotherapy was defined “durable FN.”

### Study Design and Treatments

This study included cancer patients with chemotherapy treatments that are associated with an intermediate risk of FN between 10% and 20% according to the treatment and side effect guidelines of NCCN. Therefore, all recruited patients in this study met the clinical criteria and were recommended by the NCCN for prophylactic G-CSF during chemotherapy treatment. The treatment was held for ANC <1.0×10^9^/L and/or body temperature >38°C. Given the clinical judgement and guidelines at the time of study, pegylated recombinant human G-CSF (PEG-rhG-CSF) was chosen for prophylactic use. There were four conditions for the use of antibiotic use during this study, namely, (1) decrease in ANC at grade 4, (2) occurrence of FN, (3) confirmed infection, and (4) possible infectious fever (≥38°C). For each participant, 48 h after each cycle of chemotherapy, the liquid form of PEG-rhG-CSF [Jinyouli^®^, CSPC Baike (Shijiazhuang) Biological Pharmaceutical Co., Ltd., China] with 3 mg (half dose) was administrated once *via* a subcutaneous injection. The minimum duration for each participant is two cycles of chemotherapy with prophylactic use of G-CSF. The primary endpoint was the incidence frequency of grade 3/4 neutropenia (ANC < 1.0 × 10^9^/L) at each of four cycles, and the secondary endpoints were (1) duration and incidence of all grades of FN at each cycle, (2) frequency of dose-adjusted or delay of chemotherapy at each cycle, and (3) rate of antibiotic use during chemotherapy. The start date of this study was April 5, 2018, and end date of the follow-up was August 25, 2020. For each participant, we collected clinical and demographic data including age, gender, height, weight, diagnosis, cancer type, comorbidity, type of surgery, and type of chemotherapy. Treatment-related data and blood laboratory findings were collected within 12 h prior to each prophylactic use of G-CSF, which was applied 48 h after each cycle of chemotherapy. These data include white blood cell count (WBC), neutrophil count (ANC), blood platelet count (BPC), hemoglobin count (HC), and laboratory measurements such as alanine transaminase (ALT), aspartate transaminase (AST), total bilirubin, urea nitrogen, creatinine, and estimated glomerular filtration rate (eGFR). The safety of the treatment was monitored by assessment of all adverse events with the Common Terminology Criteria for Adverse Events (CTCAE) version 5.0. We developed a smartphone-based APP program to ensure that the study protocol was carried out in a consistent and standardized way across hospitals. The principal investigators from participating hospitals were responsible for assessing outcomes. Outcomes were assessed *via* diverse ways including medical record review, personal interviewer, medical consultants, and others.

### Statistical Analysis

An optimal design with a one-sided type I error of 2.5% and 80% of power was utilized. The null hypothesis was that clinical incidence of neutropenia of III/IV degrees will be more than 30% during the treatment course (17, 18). Consequently, 151 participants were recruited in the present study. Descriptive analysis of the variables were expressed as median [interquartile range (IQR)] or number (%). Categorical data were presented as absolute and relative frequencies and compared using the chi-square test or the Fisher’s exact test. Continuous data were compared using Student’s t-test. All statistical tests were performed using R (3.6.3).

## Results

### Patients and Tumor Characteristics

**Figure 2:**
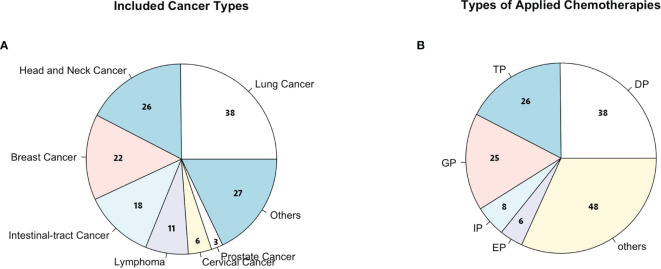
**(A)** Cancer types considered in this study; others include colorectal cancer, bladder cancer, ureter cancer, liver cancer, and gastric cancer. **(B)** Chemotherapies considered in this study; others include TP (paclitaxel + cisplatin), DP (docetaxel + cisplatin), GP (gemcitabine + cisplatin), IP (irinotecan + cisplatin), EP (etoposide + cisplatin), and FOLFOX (oxaliplatin + calcium folinate + 5-fluorouracil).

A total of 151 cancer patients [median age, 54 years old (range, 46.0–62.5]; 38.4% female] were enrolled in this study from one of these 11 hospitals in China (**Table 1**). Among this cohort, the majority of patients had lung cancer [38 (25.2%)], head and neck cancer [26 (17.2%)], breast cancer [22 (14.6%)], and esophageal carcinoma [18 (11.9%); **Figure 2A**]. For the chemotherapy regimen, the majority of patients received DP [38 (25.2%)], TP [26 (17.2%)], GP [25 (16.6%)], and EP [15 (9.9%); **Figure 2B**). According to the NCCN guidelines, all patients were estimated to have an intermediate risk of FN combined with at least one patient-specific risk factors during chemotherapy. Thus, all patients received standard doses of chemotherapy and half dose (3 mg) of PEG-rhG-CSF as a prophylactic use. There were a total of 604 cycles of chemotherapy with one half dose of PEG-rhG-CSF administration prior to each cycle.

### FN and Grade 3/4 Neutropenia

A total of five (3.3%) patients developed five episodes of FN; specifically three episodes of FN occurred in the first cycle of chemotherapy and the remaining two in the second and the third cycle, respectively. **Table 2** summarizes the duration of these FNs. Non-febrile grade 3/4 neutropenia was observed in other four (2.6%) patients in the second and third cycle of chemotherapy (**Table 2**). All of these nine (6.0%) patients reached the primary endpoints and therefore exited the study. The follow-up showed that their conditions returned to a normal level after a median duration of 13 days (95% CI, 10–17 days). After the first cycle of chemotherapy, compared to prior-treatment condition, there were significant increases in WBC [6.69 × 10^9^/L (IQR, 4.99–8.58) vs. 5.94 × 10^9^/L (IQR, 4.61–7.20); p=.001], ANC (4.30 × 10^9^/L (3.00–5.87) vs. 3.59 × 10^9^/L (IQR, 2.68–4.86); p<.001], and BPC [280 (IQR, 222–353) vs. 250 (IQR, 204–307); p=.004] in this cohort. No significant changes in WBC, ANC, and BPC were observed in this cohort during other cycles of chemotherapy.

**Table 2:** Reasons and time points of nine patients exited the study.

Cycle numbers	Reasons for exit	No. (%) of Patients
First	FN	3 (2)
Second	FN	1 (0.7)
Second	Non-febrile grade 3/4 neutropenia	2 (1.3)
Third	FN	1 (0.7)
Third	Non-febrile grade 3/4 neutropenia	2 (1.3)

### Delay, Dose-Adjusted, or Early Termination of Chemotherapy

Chemotherapy delay occurred in six (4.0%) patients for 12 (2.0%) cycles. Three of these six patients had a fever, two had intestinal complaints, and the remaining one had to change the agent of chemotherapy (**Table 3**). All causes of this treatment delay were resolved within 1 week. Dose adjustment occurred in 10 (6.6%) patients. Three (2.0%) of these 10 patients experienced reduced kidney function, 5 (3.3%) had strong decrease in physical strength, and 2 (1.3%) had grade 4 myelosuppression (**Table 3**). Early termination of cancer treatment occurred in 11 (7.3%) patients. Among them, four (2.6%) patients had neutropenia, four (2.6%) had thrombocytopenia, and three (2.0%) had sudden death.

**Table 3:** Delay, dose-adjusted, and early termination of chemotherapy(N = 25).

Cycle numbers	Cause	Reasons	No. (%) of patients
1	Treatment delay	Fever	3 (2)
1	Treatment delay	Complaint of intestine	2 (1.3)
2	Treatment delay	Change of chemotherapy agent	1 (0.7)
3	Dose-adjusted	Decrease of physical strength	5 (3.3)
3	Dose-adjusted	Myelosuppression	2 (1.3)
3	Dose-adjusted	Impaired kidney function	3 (2)
2	Early termination	Neutropenia	2 (1.3)
3	Early termination	Thrombocytopenia	4 (2.6)
3	Early termination	Sudden death	3 (2)

### Percentage of Antibiotic Use During Chemotherapy

In this cohort, 23 (15.2%) patients required antibiotic use during courses of chemotherapy. The conditions for the application of antibiotics were determined and discussed by the treating physicians. After antibiotic use, all of these patients recovered without complication and could proceed with chemotherapy.

### Adverse Effects

A total of 28 (18.5%) patients experienced clear adverse effects during cancer treatment. Among them, 24 (15.9%) patients experienced vomiting, fatigue, nausea, hair loss, and intestinal complaint that were usually associated with multiple cycle of chemotherapy. All of these adverse effects were self-limited, and afflicted patients could return to a normal condition after pause of chemotherapy. Ten (6.6%) patients had bone pain, the most common adverse effect, caused by G-SCF drugs; five (3.3%) had myalgias, three (2.0%) had headache, and three (2.0%) had dyspnea.

## Discussion

FN is the major factor contributing to substantial morbidity, mortality, and cost for patients with malignancies during chemotherapy. Studies show that approximately 25%–40% of patients receiving conventional chemotherapy will develop FN (17, 18), although diverse risk factors for FN have been reported including older age (>65 years), previous chemotherapy or radiotherapy, pre-existing neutropenia or tumor involvement, poor performance status, and comorbidities (19). G-CSF related drugs have the ability to induce proliferation and maturation of neutrophils for the reduction in the incidence of FN, which present a therapeutic support strategy to improve patient compliance and guarantee patient safety during the course of intense dose chemotherapy. Therefore, in the past several years, both NCCN and the Chinese Society of Clinical Oncology developed guidelines to provide clinical recommendations for rational use of PEG-rhG-CSF for the prevention of FN (20, 21). Although several studies investigated the clinical efficacy and safe profiles of PEG-rhG-CSF with a full dose for the reduction in FN in patients of breast or other cancers, which resulted in significant low incidence rate of FN (<2.0%) (22–24), the clinical efficacy of a half dose for cancer patients with an intermediate risk of FN remains unknown.

The present study demonstrated that the prophylactic PEG-rhG-CSF with a half dose (3 mg) could provide cancer patients sufficient benefits to prevent FN who were at an intermediate risk of FN combined with at least one patient-specific risk factor and received multiple-cycle of chemotherapy. The incidence rate of FN is 3.3%; however, none of the recruited patients required hospitalization due to FN. In line with the results of the present study, a recent study reported a favorable clinical outcome of prophylactic pegfilgrastim with a half dose for patients having a WBC count ≥10 × 10^9^/L prior to treatment cycle and discussed possible clinical criteria for this type of dose reduction (22). In another recent study, cancer patients with a weight of ≤45 and >45 kg received a half- and full dose of PEG-rhG-CSF, respectively, during cancer treatment, and its results showed a reduced FN incidence rate at 5.7% (25). Thus, our results suggest that cancer patients with a weight of >45 kg may also well-receive a half dose of PEG-rhG-CSF as long as they are at an intermediate risk of FN combined with at least one patient-specific risk factor.

Although many of G-CSF drugs were shown to be relatively safe, the major adverse effect of prophylactic use of these G-CSF-related drugs were bone pain (26, 27), bleomycin-induced pulmonary toxicities (28), and other toxicities (29), which could dramatically prolong/delay cancer chemotherapy, increase the treatment cost, and even jeopardize clinical outcome. In our study, only 10 (6.6%) patients experienced PEG-rhG-CSF-associated adverse effect, indicating a well-tolerated effect of the half dose of PEG-rhG-CSF. The price of half dose of PEG-rhG-CSF was more economic than any full dose of available G-CSF-related drugs on market. In the past, according to the NCCN guidelines, cancer patients with a high risk or an intermediate risk of FN combined with ≥1 patient-specific risk factor were recommended to use a full dose of G-CSF to prevent FN during chemotherapy. Based on our findings, we suggest that cancer patients with an intermediate risk of FN combined with ≥1 patient-specific risk factor should be adjusted with a half dose for prophylactic use of G-CSF-related drugs. This study has limitations. First, we only investigated the efficacy and safety of prophylactic use of PEG-rhG-CSF with a half dose on cancer patients with an intermediate risk. It lacked a control group of cancer patients with the same risk level receiving a full dose of PEG-rhG-CSF. Phase III clinical trials are warranted to further validate this finding in a large and randomized cohort.

## Conclusion

The prophylactic PEG-rhG-CSF with a half dose (3 mg) is shown to be efficacious and safe to prevent neutropenia for patients with diverse cancer types and an intermediate risk of FN combined with ≥1 patient-specific risk during intense dose of multiple cycle of chemotherapy.

## Data Availability Statement

The raw data supporting the conclusions of this article will be made available by the authors, without undue reservation.

## Ethics Statement

The studies involving human participants were reviewed and approved by the institutional ethics board of Tongji Hospital of Tongji Medical College, Huazhong University of Science and Technology (No. ChiECRCT-20180146). The patients/participants provided their written informed consent to participate in this study.

## Author Contribution

QM, XL, RKW, KQ, and YC: contributed to data collection and drafting the manuscript. WC, YD, ZH, JUL and JIL: contributed to statistical analysis. JUL, ML, XT, and XW: contributed to data collection. XX, BY, YZ, and RW: contributed to data preparation and analysis. JIL, GH, and QH: contributed to conceptualization.

## Funding

The Public Health and Family Planning Research Project of Hubei Province (No. WJ2019M128), Natural Science Foundation of Hubei Province (No. 2019CFB449), and General Program of National Natural Science Foundation of China (No. 81372664).

## Conflict of Interest

The authors declare that the research was conducted in the absence of any commercial or financial relationships that could be construed as a potential conflict of interest.

## Publisher’s Note

All claims expressed in this article are solely those of the authors and do not necessarily represent those of their affiliated organizations, or those of the publisher, the editors and the reviewers. Any product that may be evaluated in this article, or claim that may be made by its manufacturer, is not guaranteed or endorsed by the publisher.
